# Effect of meteorological factors and geographic location on methicillin-resistant *Staphylococcus aureus* and vancomycin-resistant enterococci colonization in the US

**DOI:** 10.1371/journal.pone.0178254

**Published:** 2017-05-30

**Authors:** Natalia Blanco, Eli Perencevich, Shan Shan Li, Daniel J. Morgan, Lisa Pineles, J. Kristie Johnson, Gwen Robinson, Deverick J. Anderson, Jesse T. Jacob, Lisa L. Maragakis, Anthony D. Harris, for the CDC Prevention Epicenter Program

**Affiliations:** 1 Department of Epidemiology and Public Health, University of Maryland School of Medicine, Baltimore, United States of America; 2 Department of Internal Medicine, University of Iowa College of Medicine, Iowa City, United States of America; 3 Department of Biostatistics, Indiana University Fairbanks School of Public Health, Indianapolis, United States of America; 4 VA Maryland Healthcare System, Baltimore, United States of America; 5 Department of Pathology, University of Maryland School of Medicine, Baltimore, United States of America; 6 Department of Medicine, Duke University School of Medicine, Durham, United States of America; 7 Division of Infectious Diseases, Department of Medicine, Emory University School of Medicine, Atlanta, United States of America; 8 Department of Medicine, Division of Infectious Diseases, Johns Hopkins University School of Medicine, Baltimore, United States of America; Centre Hospitalier Universitaire Sainte-Justine, CANADA

## Abstract

**Background:**

Little is known about the effect of meteorological conditions and geographical location on bacterial colonization rates particularly of antibiotic-resistant Gram-positive bacteria. We aimed to evaluate the effect of season, meteorological factors, and geographic location on methicillin-resistant *Staphylococcus aureus* (MRSA) and vancomycin-resistant enterococci (VRE) colonization.

**Methods:**

The prospective cohort included all adults admitted to 20 geographically-dispersed ICUs across the US from September 1, 2011 to October 4, 2012. Nasal and perianal swabs were collected at admission and tested for MRSA and VRE colonization respectively. Poisson regression models using monthly aggregated colonization counts as the outcome and mean temperature, relative humidity, total precipitation, season, and/or latitude as predictors were constructed for each pathogen.

**Results:**

A total of 24,704 ICU-admitted patients were tested for MRSA and 24,468 for VRE. On admission, 10% of patients were colonized with MRSA and 12% with VRE. For MRSA and VRE, a 10% increase in relative humidity was associated with approximately a 9% increase in prevalence rate. Southerly latitudes in the US were associated with higher MRSA colonization, while northerly latitudes were associated with higher VRE colonization. In contrast to MRSA, the association between VRE colonization and latitude was observed only after adjusting for relative humidity, which demonstrates how this effect is highly driven by this meteorological factor.

**Conclusions:**

To our knowledge, we are the first to study the effect of meteorological factors and geographical location/latitude on MRSA and VRE colonization in adults. Increasing humidity was associated with greater MRSA and VRE colonization. Southerly latitudes in the US were associated with greater MRSA and less VRE. The effect of these factors on MRSA and VRE rates has the potential not only to inform patient management and treatment, but also infection prevention interventions.

## Background

Throughout history, certain infectious diseases have been tightly correlated with seasonal, meteorological, and climatic conditions [1]. The winter peaks in influenza infections are an example of this interaction. However, the mechanisms underlying this association, particularly on pathogens transmitted from person-to-person, are not yet well understood [1]. Furthermore, despite the global public health importance of infections associated with Gram-positive bacteria [2], few studies have examined its association with seasonal and meteorological conditions and yielded inconsistent results [3–8].

Patients can be either infected or colonized with bacteria. Colonization is the presence of bacteria in an anatomic site without any symptoms of disease [9–11]. Colonization is detected by obtaining surveillance cultures, while bacterial infection is identified through clinical cultures after signs and symptoms are evident, often at sites other than the site of colonization. As bacterial colonization represents an earlier step in the disease pathway, research at this level can provide valuable insights. However, studies evaluating the correlation between seasonal and meteorological factors with methicillin-resistant *Staphylococcus aureus* (MRSA) and vancomycin-resistant enterococci (VRE) colonization are limited and have focused on infant populations, particularly on neonates (Table 1) [12,13].

**Table 1:** Relevant and related studies on the effect of meteorological factors and geographical location on MRSA and VRE colonization rates.

	Sites	Population	Study period	Colonization vs. infection	Study objective	Related statistical analysis	Related findings
**MRSA (*S*.*aureus*)**							
Ogawa, 1994 [14]	Single	Children and adults	June to August 1993	Colonization or infection	To compare Staphylococcal flora on the skin surface of atopic dermatitis patients and healthy subjects.	Student’s T test	-A significant seasonal difference on *S*. *aureus* carriage in the forearms of atopic dermatitis patients was observed. -More *S*. *aureus* carriage was observed in the summer compared to winter.
Harrison, 1999 [15]	Single	Children	One year	Colonization	To determine the effect of age, gender, season, viral upper respiratory tract infection, and sleeping position on the composition of the nasopharyngeal flora in infancy.	Chi square	-No significant association between seasonality and *S*. *aureus* carriage was observed. -More carriage was observed in autumn/winter months.
Kaier, 2010[16]	2 large university hospitals in Germany	N.S	January 2005 to May 2009	Colonization and infection	To determine whether there was seasonality in the incidence of extended-spectrum β-lactamase-producing bacteria and MRSA carriage.	Time-series analysis	-No association between MRSA and temperature was detected.
Eber, 2011 [3]	132 US hospitals	N.S	January 1999 to September 2006	Infection	To evaluate seasonal changes in the frequencies of BSIs.	-Time-series analysis: models were adjusted by the nine US Census Bureau regional divisions	-No significant difference across seasons for *S*. *aureus* was observed. -An increase of 5.6°C (10°F) was associated with an adjusted increase of 2.2% in frequency of *S*. *aureus* associated BSIs. -A one-inch increase in monthly precipitation was associated with 0.3% lower frequency of *S*. *aureus* associated BSIs. -No significant association between *S*. *aureus* and humidity.
Perencevich, 2008 [4]	University of Maryland Medical Center	Adults	January 1998 to December 2005	Infection	To assess whether seasonal variation existed in incidence of infection and to quantify the relationship between temperature changes and infection rates.	-Time-series analysis	-No summer peaks for *S*. *aureus* were observed. -No association between temperature and *S*.*aureus* was described.
Klein, 2013 [17]	*S*.*aureus* isolates across US inpatients	Children and adults	January 2005 to December 2008	Infection	To estimate the incidence and patterns of hospital-acquired (HA) -MRSA and community-acquired (CA)-MRSA-related hospitalizations, as well as the influence of seasonal variations.	-Seasonal trend decomposition method.	-CA-MRSA incidence peaked in late summer, particularly in children. -HA-MRSA incidence peaked in the winter.
Wang, 2013 [18]	Maricopa County, Arizona	Children	January 2005 to December 2008	Infection	To determine the temporal trend, seasonality pattern, and peak timing of MRSA infections in different children’s age groups.	-Time-series analysis and non-linear regression analysis	-A strong annual seasonal pattern of skin and soft tissue infection (SSTI) incidence was observed with peaks occurring in September. -A significant direct correlation between SSTI incidence and mean temperature. was observed. The same was observed for humidity.
Schwab, 2014 [5]	73 German ICUs	Adult	January 2001 to December 2012	Colonization and infection	To look for temperature associations with pathogens in a network of geographically variant sites.	-Time series analysis: location was not included in the models.	-An increase of 5°C during the prior month to isolation was associated with a 1% decrease of *S*. *aureus*.
Sahoo, 2014 [8]	Katalinga Institute of Medical Science in India	Children and adults	July 2009 to December 2010	Infection	To analyze the association of *S*. *aureus* and MRSA SSTI with local temperature and relative humidity	-Time-series analysis	-An increase of 1.7°C in maximum temperature and a 10% increase in RH was associated with one unit increase in MRSA occurrence.
Giuffre, 2015 [12]	1 NICU in Italy	Neonates	June 2009 to June 2013	Colonization	To describe epidemiologic features and identify risk factors for MRSA acquisition in a level III Neonatal ICU.	-Chi square	-A seasonal variation was evident for MRSA colonization with incidence density peaking in the summer and autumn quarters (June-November).
Albernoor, 2016 [19]	97 cohort studies	Adults	-	Infection	To summarize the frequency of mediastinitis following open-heart surgery caused by Gram-positive bacteria and the effect of several moderator variables including latitude	-Meta-analysis, meta-regression models	-A negative association between the frequency of mediastinitis and latitude of study site was observed.
**VRE**							
Dauner, 2000 (abstract) [20]	Hospitals, physicians and/or laboratories in Arizona	Adults and children	January 1998 to December 1999.	Colonization and infection	To determine age and county specific incidence rates for VRE	-Estimation of age and county specific incidence rates for VRE	-No seasonal variation was observed in either year.
Hufnagel, 2007 [13]	1 NICU in Germany	Neonates	March 2003 to February 2004	Colonization	To analyze predictors for early enterococcal colonization of infants in a NICU and to describe risk factors associated with multidrug resistant enterococci colonization and its seasonal patterns.	Chi-square, logistic regression	-A significantly higher number of *Enterococci* and multi-drug resistant *enterococci* was observed during winter/spring months.

N.S. = Not specified in the abstract/manuscript

Additionally, our understanding of the effect of geographic location (latitude) on MRSA and VRE rates is limited. Latitude represents not only geographical location, but it can also act as a proxy of environmental conditions, in addition to differences in topography, access and quality of healthcare, and socio-economic conditions. Thus, latitude allows us to explore not only the effect of geographic location but also if this effect is fully explained by meteorological conditions or if some other factors should be studied. The previous effects have not been fully explored for VRE and MRSA effect as multi-site studies on this topic are rare [3,5,16].

To further explore these gaps, this study aims to address two different research questions. First, we aim to evaluate the effect of season, temperature, humidity, and precipitation on MRSA and VRE colonization among adults. Second, we aim to assess and explore the effect of geographical location on MRSA and VRE colonization. To our knowledge, we are the first to address these questions specifically on MRSA and VRE adult colonization. Understanding these associations has the potential not only to inform patient management and treatment, but also infection prevention interventions. This knowledge can apprise local hospital infection preventionists and/or state and national public health authorities if resources and preventive measures should be heightened during certain times of the year or in certain geographical locations year around.

## Methods

### Population

**Fig 1:**
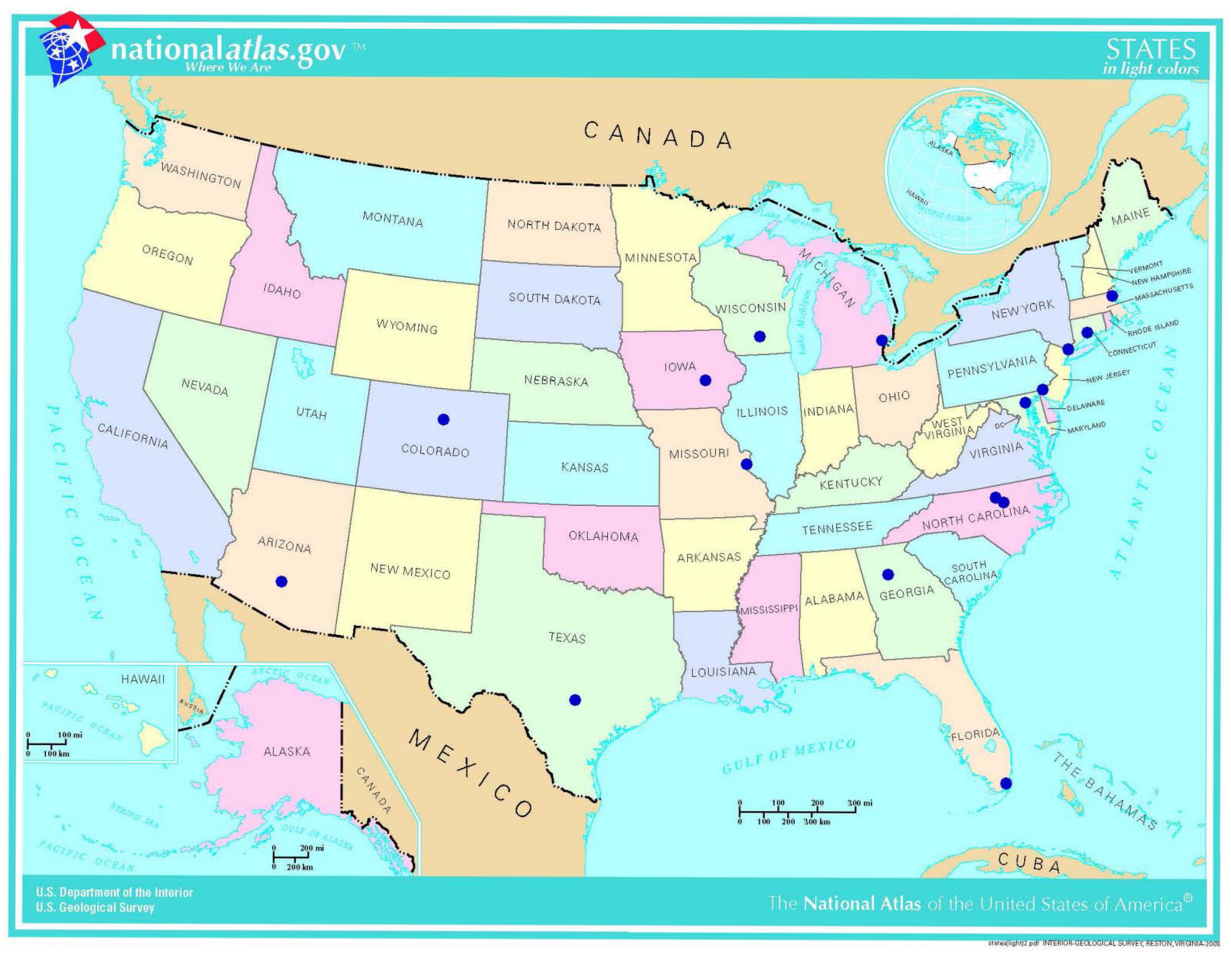
Distribution of study cities. The BUGG study included 20 ICU-sites across 16 cities across the US.

We analyzed a prospective cohort of adult patients admitted to 20 geographically-dispersed intensive care units (ICUs) across the US as part of the Benefits of Universal Glove and Gown (BUGG) cluster-randomized trial during the period between September 1, 2011 and October 4, 2012 [21] (Fig 1). For the purpose of this analysis, nasal and perianal admission swabs were tested for MRSA and VRE respectively using culture and PCR to detect resistance genes (*mecA* or *vanA*/*vanB*). The University of Maryland School of Medicine served as the central institutional review board (IRB) for the BUGG study. All participating ICUs (University of Maryland Medical Center, Barnes Jewish Hospital, Boston Medical Center, Brigham and Women's Hospital, Christiana Hospital, Denver Medical Center, Duke University Hospitals (Durham and Raleigh), Emory University Hospital, Henry Ford Hospital, Jackson Memorial Hospital, John Hopkins Hospital, Lawrence and Memorial Hospital, Weill Cornell Medical College, St. Luke’s Medical Center, University of Iowa Hospitals, University of Miami, University of Texas Health Science Center, University of Wisconsin Hospital and Clinics, Wake Med Hospital) received approval from their local IRBs, and each determined this to be a minimal-risk study and granted approval of the study along with a waiver of consent and Health Insurance Portability and Accountability Act (HIPAA) waiver (Trial registration number (clinicaltrials.gov Identifier): NCT0131821).

### Meteorological and geographical variables

We collected mean monthly temperature (°F), mean monthly relative humidity (%), and monthly total precipitation (inches) by city from the National Oceanic and Atmospheric Administration website [22]. In addition, latitude from each hospital site was collected from the *My NASA Data* website [23]. The previous variables were included in the analysis as continuous variables (Table 2). In addition, a categorical variable for season was created based on the swab collection date as follows: winter (December to February), spring (March to May), summer (June to August), and autumn (September to November).

**Table 2:** Latitude and average of monthly meteorological variables per season by study site, September 2011 to October 2012.

Site	Latitude (°)	Season	N*		Mean (SD)		
			MRSA	VRE	Mean temperature (°F)	Total precipitation (Inches)	Relative humidity (%)
University of Maryland, Baltimore, MD	39.29	Winter	149	150	42.87 (2.36)	2.13 (0.72)	64.00 (4.36)
		Spring	267	266	61.33 (8.55)	2.00 (0.71)	62.33 (8.62)
		Summer	231	228	79.95 (3.83)	3.64 (1.39)	66.00 (6.00)
		Fall	329	329	63.09 (8.40)	4.54 (2.55)	74.40 (5.18)
Barnes Jewish, St. Louis, MO	38.64	Winter	108	108	39.83 (1.97)	2.51 (0.83)	64.67 (3.79)
		Spring	186	186	64.27 (7.28)	3.93 (2.95)	57.33 (3.79)
		Summer	192	192	80.93 (5.18)	2.32 (1.98)	50.33 (3.21)
		Fall	228	229	60.41 (7.42)	3.47 (1.14)	62.20 (3.35)
Boston Medical Center, Boston, MA	42.34	Winter	123	123	37.23 (2.95)	1.81 (1.09)	59.67 (5.13)
		Spring	162	162	53.33 (6.85)	1.47 (0.65)	62.67 (12.01)
		Summer	199	199	72.27 (4.83)	2.49 (1.24)	67.67 (2.31)
		Fall	223	221	59.32 (6.79)	2.71 (1.25)	70.40 (4.72)
Brigham & Women’s Hospital, Boston, MA	42.34	Winter	228	227	37.23 (2.95)	1.81 (1.09)	59.67 (5.13)
		Spring	381	376	53.33 (6.85)	1.47 (0.65)	62.67 (12.01)
		Summer	382	380	72.27 (4.83)	2.49 (1.24)	67.67 (2.31)
		Fall	511	511	59.32 (6.79)	2.71 (1.25)	70.40 (4.72)
Christiana Hospital, Newark, DE	39.69	Winter	262	261	40.03 (2.83)	3.11 (1.94)	.
		Spring	447	444	57.63 (7.84)	2.55 (0.81)	.
		Summer	408	408	76.87 (4.29)	3.33 (1.41)	.
		Fall	502	501	61.68 (8.48)	5.72 (1.95)	.
Denver Medical Center, Denver, CO	39.73	Winter	172	168	31.23 (4.39)	1.42 (0.88)	57.33 (8.96)
		Spring	345	342	54.34 (5.72)	0.86 (0.74)	40.00 (7.21)
		Summer	321	320	75.41 (2.10)	1.10 (0.94)	33.00 (4.36)
		Fall	439	436	54.25 (10.43)	1.30 (0.56)	43.40 (3.97)
Duke University, Durham, NC	36.01	Winter	213	209	44.30 (1.01)	1.82 (0.20)	64.00 (2.00)
		Spring	301	300	61.35 (6.28)	3.86 (1.27)	68.00 (4.58)
		Summer	332	329	76.38 (4.64)	3.60 (1.08)	70.33 (6.11)
		Fall	371	364	61.35 (7.90)	4.37 (2.15)	71.80 (3.56)
Emory University Hospital Midtown, Atlanta, GA	33.79	Winter	247	239	49.30 (1.37)	3.17 (1.49)	63.67 (3.06)
		Spring	416	402	67.20 (5.15)	2.75 (1.03)	61.33 (3.21)
		Summer	426	417	79.15 (3.38)	3.28 (0.75)	65.67 (5.03)
		Fall	492	479	64.59 (7.96)	2.35 (0.56)	63.40 (2.88)
Henry Ford, Detroit, MI	42.37	Winter	154	154	33.21 (2.36)	2.07 (0.60)	74.00 (2.00)
		Spring	280	279	55.09 (8.81)	2.20 (0.44)	60.67 (4.04)
		Summer	323	320	74.77 (3.57)	2.52 (1.33)	59.67 (4.51)
		Fall	322	317	56.19 (7.84)	4.09 (2.27)	70.00 (4.00)
Jackson Memorial Hospital, Miami FL	25.79	Winter	187	185	70.69 (2.59)	1.98 (2.52)	68.33 (4.51)
		Spring	307	307	76.13 (2.44)	6.44 (3.93)	68.33 (4.93)
		Summer	290	290	82.60 (0.80)	9.90 (2.40)	73.67 (1.15)
		Fall	409	407	79.50 (3.39)	7.63 (4.85)	73.00 (2.24)
John Hopkins Hospital, Baltimore, MD	39.30	Winter	147	145	42.87 (2.36)	2.13 (0.72)	64.00 (4.36)
		Spring	209	205	61.33 (8.55)	2.00 (0.71)	62.33 (8.62)
		Summer	257	254	79.95 (3.83)	3.64 (1.39)	66.00 (6.00)
		Fall	329	327	63.09 (8.40)	4.54 (2.55)	74.40 (5.18)
Lawrence & Memorial Hospital, New Haven, CT	41.34	Winter	114	112	37.50 (2.46)	2.70 (1.12)	.
		Spring	201	201	53.67 (7.83)	3.22 (2.04)	.
		Summer	175	171	73.53 (3.79)	4.32 (0.91)	.
		Fall	254	250	59.60 (8.14)	4.78 (1.66)	.
Weill Cornell Medical College, New York, NY	40.76	Winter	185	185	40.41 (2.82)	2.55 (1.19)	59.00 (3.61)
		Spring	302	301	56.61 (7.61)	3.33 (2.34)	60.67 (12.58)
		Summer	266	264	76.08 (3.95)	3.63 (0.91)	65.33 (2.08)
		Fall	373	368	61.65 (8.03)	4.43 (1.80)	70.60 (5.32)
St. Luke's Medical Center, Phoenix, AZ	33.45	Winter	159	155	54.65 (3.55)	0.36 (0.62)	39.67 (11.55)
		Spring	220	216	72.15 (9.32)	0.19 (0.18)	19.67 (5.03)
		Summer	233	228	91.83 (1.21)	0.84 (0.82)	27.00 (11.27)
		Fall	267	256	77.36 (10.99)	0.36 (0.41)	30.40 (8.62)
University of Iowa, Iowa City, IA	41.66	Winter	459	458	31.53 (2.14)	1.31 (1.04)	.
		Spring	756	750	58.42 (8.08)	3.32 (1.37)	.
		Summer	797	791	76.20 (5.21)	2.02 (1.80)	.
		Fall	986	972	54.95 (8.60)	2.42 (0.71)	.
University of Miami, Miami, FL	25.79	Winter	120	118	70.69 (2.59)	1.98 (2.52)	68.33 (4.51)
		Spring	176	176	76.13 (2.44)	6.44 (3.93)	68.33 (4.93)
		Summer	207	208	82.60 (0.80)	9.90 (2.40)	73.67 (1.15)
		Fall	250	248	79.50 (3.39)	7.63 (4.85)	73.00 (2.24)
University Hospital. San. Antonio, TX	29.51	Winter	249	249	55.05 (1.38)	3.27 (0.64)	69.67 (5.86)
		Spring	408	406	72.59 (7.16)	3.09 (3.21)	70.33 (5.03)
		Summer	376	376	85.34 (1.22)	2.19 (1.74)	62.67 (4.04)
		Fall	542	539	73.47 (8.03)	3.21 (2.14)	61.60 (9.29)
University of Wisconsin Hospital & Clinics, Madison, WI	43.08	Winter	282	281	28.63 (2.76)	1.61 (0.83)	72.33 (2.31)
		Spring	451	446	53.77 (8.34)	2.80 (0.71)	63.67 (4.04)
		Summer	563	556	73.87 (4.73)	2.11 (1.85)	60.00 (6.00)
		Fall	623	616	52.12 (8.31)	2.19 (1.05)	68.80 (3.83)
Wake Med Hospital, Raleigh, NC	35.78	Winter	127	127	47.13 (1.26)	1.91 (0.32)	64.00 (2.00)
		Spring	156	155	64.03 (6.82)	3.48 (1.30)	68.00 (4.58)
		Summer	178	178	79.02 (4.58)	4.29 (1.66)	70.33 (6.11)
		Fall	233	231	64.08 (8.26)	4.28 (2.12)	71.80 (3.56)
Duke University, Raleigh, NC	35.83	Winter	199	194	47.13 (1.26)	1.91 (0.32)	64.00 (2.00)
		Spring	319	315	64.03 (6.82)	3.48 (1.30)	68.00 (4.58)
		Summer	341	336	79.02 (4.58)	4.29 (1.66)	70.33 (6.11)
		Fall	350	339	64.08 (8.26)	4.28 (2.12)	71.80 (3.56)

* N represents total number of patients tested for either MRSA or VRE by site per season.

### Statistical analysis

#### Descriptive analysis

Monthly proportions of MRSA or VRE colonization on admission were initially estimated per site. We estimated correlation coefficients between these proportions and each meteorological variable. We also performed tests of proportions (Generalized linear model (GLM)) across sites and seasons.

#### Univariate models

To determine the effect of each meteorological and geographical variable on colonization, we first constructed a Poisson regression model for each pathogen using monthly aggregated colonization counts per site as its outcome and mean temperature, relative humidity, total precipitation, season, or latitude as the primary exposure as individual variables. The log of the total monthly number of swabs collected and processed per site was defined as the offset of the model. Initially, we detected overdispersion, usually due to higher variability among counts than would have been expected for a Poisson distribution, which affected our models’ goodness of fit (deviance and Pearson Chi Square) [24]. We accounted for overdispersion by introducing a dispersion parameter to the model [24]. The statistical methods chosen were consistent with prior studies [4,25].

#### Multivariate models

Model 1: Combined effect of meteorological variables. This multivariate model evaluated the association between the monthly prevalence of each pathogen and meteorological variables (mean temperature, relative humidity, and precipitation).

Model 2: Effect of geographical location. This multivariate model evaluated the association between the monthly prevalence of each pathogen and the ICU’s geographical location (latitude), adjusting for the meteorological variables that were statistically significant (p<0.05) in their respective univariate models.

## Results

### Descriptive analysis

Our study was conducted in 20 different ICUs across 16 different US states with an average of 19 beds (range: 9–36 beds) per ICU. Fifty five percent of our ICUs were medical intensive care units (MICUs), while the rest were surgical intensive care units (SICUs) (25%) or a combination of both (MICU/SICU) (20%). On average, a total of 1223 patients per hospital ICU were tested for VRE (range: 691–2971) and 1235 patients were tested for MRSA (range: 694–2998) [21]. In summary, a total of 24,704 and 24,468 patients were tested for MRSA and VRE colonization respectively upon admission to the ICU. Overall, we observed an average of 10% MRSA colonization on admission, ranging from 3% to 16% for each hospital. For VRE, we observed an average of 12% colonization rate on admission, ranging from 3% to 25%.

In our dataset, mean temperature was positively correlated with total precipitation (r = 0.34, p<0.001). Similarly, relative humidity was positively correlated with total precipitation (r = 0.54, p<0.001). However, no significant association was observed between temperature and relative humidity. In contrast, latitude was negatively correlated with mean temperature (r = -0.48, p<0.001), total precipitation (r = -0.38, p<0.001), and relative humidity (r = -0.06, p = 0.379).

MRSA monthly colonization was positively correlated with mean temperature (r = 0.16, p = 0.008), relative humidity (r = 0.24, p<0.001), and total precipitation (r = 0.22, p<0.001). MRSA monthly colonization was negatively correlated with latitude (r = -0.34, p<0.001) i.e. colonization was higher at latitudes closer to the equator. For VRE, a positive correlation was only observed between VRE monthly colonization and relative humidity (r = 0.20, p = 0.004). In contrast, for VRE, no significant correlations were detected with total precipitation, mean temperature, or latitude. No significant difference on MRSA or VRE colonization was observed across seasons (p = 0.589 and p = 0.922 respectively).

### Univariate models

For MRSA, positive associations were observed in our univariate Poisson models between MRSA counts and all studied meteorological conditions. For every unit (°F) increase of mean monthly temperature, there was a 0.7% increase in MRSA prevalence (p = 0.002). Furthermore, for every 1% increase in relative humidity, there was a 1.3% increase in pFrevalence (p<0.001). Similarly, a 5.6% increase in MRSA prevalence was observed per one-inch increase in annual precipitation (p<0.001). In addition, for every one degree (°) reduction of latitude, there was a 3.8% increase in prevalence (p<0.001). However, no significant association was observed between colonization and season (p = 0.669).

For VRE, a positive association was only observed in our univariate Poisson models between VRE counts and relative humidity. For every 1% increase in relative humidity, there was a 0.9% increase of VRE prevalence (p = 0.015). In addition, no significant association was observed between colonization and season (p = 0.669) or latitude (p = 0.672).

### Multivariate models

Model 1: Combined effect of meteorological variables. For MRSA, only relative humidity remained significant in this multivariate model (Table 3). For every 1% increase of relative humidity, there was approximately a 0.9% increase in MRSA colonization when controlling for the other meteorological variables (p = 0.010).

**Table 3:** Prevalence rate ratio of MRSA colonization by monthly meteorological variables and latitude among 24, 704 ICU patients across 20 US sites, September 2011 to October 2012.

Variable	Model 1*		Model 2 **	
	Prevalence Rate Ratio	P value	Prevalence Rate Ratio	P value
**Mean temperature (**°**F)**	1.003	0.220	1.000	0.962
**Relative humidity (%)**	1.009	0.010	1.010	0.004
**Total precipitation (Inches)**	1.024	0.139	1.006	0.693
**Latitude (**°**)**	-	-	0.977	0.001

*Multivariate model included the following variables: mean temperature, relative humidity, and total precipitation.

** Multivariate model included the following variables: latitude, mean temperature, relative humidity, and total precipitation.

For VRE, only relative humidity approached significance in this multivariate model (Table 4). For every 1% increase of relative humidity, there was approximately a 0.8% increase in VRE colonization when controlling for the other meteorological variables (p = 0.064).

**Table 4:** Prevalence rate ratio of VRE colonization by monthly meteorological variables, and latitude among 24,468 ICU patients across 20 US sites, September 2011 to October 2012.

Variable	Model 1*		Model 2 **	
	Prevalence Rate Ratio	P value	Prevalence Rate Ratio	P value
**Mean temperature (**°**F)**	0.996	0.173	-	-
**Relative humidity (%)**	1.008	0.064	1.010	0.008
**Total precipitation (Inches)**	1.009	0.660	-	-
**Latitude (**°**)**	-	-	1.018	0.019

*Multivariate model included the following variables: mean temperature, relative humidity, and total precipitation.

** Multivariate model included the following variables: latitude and relative humidity.

Model 2: Effect of geographical location. For MRSA, latitude was negatively associated with colonization even after adjusting for meteorological variables (p = 0.001). A 2.4% increase in MRSA colonization was observed per unit decrease of latitude (°) (Table 3). In other words, southern states had higher MRSA colonization rates than northern states.

For VRE, latitude was positively associated to colonization after controlling for the confounding effect of relative humidity (p = 0.019). A 1.8% increase in VRE colonization was observed per unit increase of latitude (°). In other words, northern states had higher VRE colonization than southern states (Table 4).

## Discussion

We observed a significant effect of meteorological factors and geographical location on MRSA and VRE colonization in our study population. For MRSA and VRE, a 10% increase in relative humidity led to an 8–9% increase in prevalence rate. Furthermore, we observed a stronger effect of geographical location on colonization. Interestingly, the direction of this association varied by pathogen. The closer to the equator, the higher the observed MRSA colonization but the lower the observed VRE colonization.

Literature specifically studying the effect of season on MRSA and VRE colonization is very limited. There are only three studies assessing this effect specifically on MRSA colonization. Similar to our study, Ogawa et al. also reported no significant difference in *S*. *aureus* colonization across seasons on the skin of 40 healthy individuals in Japan [14]. Likewise, Harrison et al. observed no significant difference on *S*. *aureus* colonization across seasons among 72 infants in the United Kingdom [15]. In contrast, Giuffrè et al. described an incidence density peak of MRSA nasal colonization among neonates (n = 832) admitted to the neonatal ICU (NICU) during the summer and autumn quarters [12], although no statistical analysis was done. With regard to VRE, only Hufnagel et al. have reported a significant increase in multidrug resistant enterococcus colonization in winter and spring months across 274 neonates admitted to the NICU [13]. The epidemiological differences between adult and neonatal populations, such as different levels of immunity, types of exposure and risk factors (14–16), and a different microbiome that potentially could have an effect of antibiotic-resistant Gram-positive bacteria colonization (17,18), may explain the observed differences with our results.

Unlike previous studies on these pathogens, we were particularly interested to study the effect of geographic location or latitude on colonization rates. Generally, higher temperatures and lower seasonal variation can be observed closer to the equator [26]. Fisman et al. found that Gram negative-associated bacteremia is more common in locations closest to the equator [27]. Albelnoor et al. also described higher rates of MRSA-associated mediastinitis across sites with lower latitude [19]. Similarly, we observed more MRSA colonization in the southerly sites. However, the opposite was observed for VRE. Our northerly sites had higher VRE colonization rates. The appearance of the effect of latitude on VRE colonization only after adjusting for relative humidity demonstrates this effect is highly driven by this meteorological factor. To our knowledge, there are no other studies describing the effect of latitude on VRE colonization or infection rates.

Additionally in our study, the observed geographic effect particularly on MRSA colonization remains significant even after adjusting for meteorological variables. This suggests that other factors besides the analyzed meteorological variables are necessary to explain this association. Further studies are necessary to identify these specific other factors.

This study has several limitations. The most important limitation is that we only had one year of data. Hence, we were unable to perform a more rigorous time-series analysis to investigate VRE and MRSA seasonality or to further analyze the effect of meteorological factors and geographical location. We believe that future studies should include several years to confirm the observed effects on bacterial colonization. In addition, we could not adjust for important confounders. Antibiotic use/prescription has been associated with the prevalence of antibioticresistant microorganisms in a number of studies at the patient level. For instance, Sun et al. reported a correlation between prescriptions of fluoroquinolones and prevalence of ciprofloxacin-resistant MRSA [28]. Another limitation is that we were unable to collect and adjust on hospital-level confounders (i.e. age distribution, sociodemographic status) for each of the study sites.

Nevertheless, the main strength of this study was the ability to assess the effect of meteorological variables on solely MRSA and VRE colonization instead of infection (or a combination). As colonization is an earlier step in the disease pathway, research at this level may provide valuable insights to strengthen prevention strategies as colonized individuals could potentially have different risk factors than infected individuals. In addition, due to the diversity of sites across the US that made up our study, to our knowledge, we are the first to study the effect of geographical location (latitude) on MRSA and VRE adult colonization rates.

Further studies should investigate the effect of latitude using global multi-site data. Moreover, the role played by additional factors such as weather, topography, or socioeconomic factors that we were unable to collect should be investigated to help elucidate the different geographical effect observed across microorganisms. Additionally, the potential effect of global warming on bacterial colonization and infection should be analyzed.

MRSA and VRE adult colonization rates at admission to ICUs across the US are far from inconsequential and should be considered when making decisions on patient care and infection control. For example, bacterial colonization information can drive local empiric antibiotic choice and influence local infection control intervention choices. Additionally, relative humidity and geographical location appear to have an important effect on VRE and MRSA colonization rates. If our results are confirmed in future studies, this conclusion has different implications for different levels of public health. At the local level, infection preventionists could enhance surveillance and decolonization measures during humid periods regardless of season. In contrast, state and national public health officials may need to incorporate the effect of geographical location on their decision making process depending on the pathogen of interest and regardless of the time of year. For instance, they could allocate more funding for MRSA prevention to southern states than northern states year round regardless of season.
